# Hypoxia-inducible factor 1-alpha is a driving mechanism linking chronic obstructive pulmonary disease to lung cancer

**DOI:** 10.3389/fonc.2022.984525

**Published:** 2022-10-21

**Authors:** Yuan-rui Xu, An-long Wang, Ya-qing Li

**Affiliations:** ^1^ The Second Clinical Medical College, Zhejiang Chinese Medical University, Hangzhou, Zhejiang, China; ^2^ Cancer Hospital of the University of Chinese Academy of Sciences (Zhejiang Cancer Hospital), Hangzhou, Zhejiang, China

**Keywords:** chronic obstructive pulmonary disease, lung cancer, non-small cell lung cancer, hypoxia-inducible factor 1-alpha, hypoxia-inducible factor 1

## Abstract

Patients with chronic obstructive pulmonary disease (COPD), irrespective of their smoking history, are more likely to develop lung cancer than the general population. This is mainly because COPD is characterized by chronic persistent inflammation and hypoxia, which are the risk factors for lung cancer. However, the mechanisms underlying this observation are still unknown. Hypoxia-inducible factor 1-alpha (HIF-1α) plays an important role in the crosstalk that exists between inflammation and hypoxia. Furthermore, HIF-1α is the main regulator of somatic adaptation to hypoxia and is highly expressed in hypoxic environments. In this review, we discuss the molecular aspects of the crosstalk between hypoxia and inflammation, showing that HIF-1α is an important signaling pathway that drives COPD progression to lung cancer. Here, we also provide an overview of HIF-1α and its principal regulatory mechanisms, briefly describe HIF-1α-targeted therapy in lung cancer, and summarize substances that may be used to target HIF-1α at the level of COPD-induced inflammation.

## Introduction

Lung cancer is the leading cause of cancer-related death worldwide (1). Although immunotherapy has drastically improved the prognosis of lung cancer patients, its efficacy is constrained to individuals with more than 50% programmed cell death 1 ligand 1 (PD-L1) expression. Accordingly, a global multicenter study of PD-L1 expression in patients with locally advanced or metastatic non-small cell lung cancer (NSCLC) reported that only 22% of the patients expressed PD-L1 levels greater than 50% (2). NSCLC is the predominant histological subtype of lung cancer that accounts for 85% of all lung cancer cases (3). Chronic obstructive pulmonary disease (COPD), which is categorized by chronic inflammation and airflow restriction, is prevalent among patients with NSCLC and notably increases mortality. COPD occurs because individuals with COPD and lung cancer are less likely to receive chemotherapy or immunotherapy. However, if the progression of COPD to lung cancer can be delayed, the incidence and mortality rates of lung cancer will be reduced. Therefore, it is crucial to first understand how the recognized risk factors contribute to the early progression of lung cancer in order to achieve this objective.

## COPD and lung cancer are related

Smoking is a major risk factor for lung cancer, and patients with COPD are four to six times more likely to develop lung cancer than smokers without COPD (4). Furthermore, COPD may cause lung cancer in nonsmokers (4). Therefore, COPD/emphysema is a risk factor for lung cancer regardless of smoking status (5). In a study, 2,507 patients with COPD were monitored for an average of 60 months. Overall, 215 individuals developed lung cancer at a rate of 16.7 per 1,000 person-years after the follow-up (6). COPD severity is also a critical risk factor for lung cancer. In a large epidemiological study with a 22-year follow-up of 5,402 participants, an increased COPD severity was linked to an elevated risk of lung cancer (7). Moreover, emphysema diagnosed using computed tomography (CT) and airflow restriction determined using spirometry is also associated with lung cancer (8). Accordingly, annual CT screening has been demonstrated to enhance lung cancer diagnosis and decrease mortality, although this is only in individuals with normal or minimally compromised lung function (9).

Approximately 1% of COPD patients develop lung cancer each year (10, 11). According to the latest Global Burden of Disease (GBD) statistics, the number of people with COPD exceeded 1,400,000 in 2018, and the number of people with lung cancer increased by about 400,000 from 2018 to 2019 (12). Therefore, it is likely that about 30% of the new lung cancer population from 2018 to 2019 is related to COPD. It’s a staggeringly theoretical value. However, there are no real-world statistical findings to confirm this figure. In addition, smoking is known to be a common risk factor for COPD and lung cancer. Therefore, this theoretical value is not influenced by COPD alone. However, it has been shown above that the risk of lung cancer is much greater in COPD patients than in the smoking population. Therefore, this theoretical data is sufficient to warn us to pay attention to COPD among the risk factors for lung cancer.

## Inflammation and hypoxia link COPD with lung cancer

Numerous mechanisms, including chronic inflammation, aberrant immunology, oxidative stress, epithelial-mesenchymal transition, hypoxia, and genetic variables, have been used to elucidate the association between COPD and lung cancer (13–22). Hypoxia as a carcinogenic etiology of COPD has recently piqued the interest of researchers. Interestingly, a reciprocal relationship exists between hypoxia and inflammation. Hypoxia can aggravate inflammation by activating inflammatory pathways and influencing immune cell functions. Conversely, during inflammation, pathologies such as thrombosis, trauma, compression (interstitial hypertension), and atelectasis (airway obstruction) elevate cells’ metabolic requirements, consequently decreasing oxygen delivery and worsening tissue hypoxia (23). Therefore, hypoxia and inflammation significantly contribute to COPD carcinogenesis, and HIF-1α plays a critical role in both processes. Additionally, hypoxia can increase HIF-1α expression in hypoxic tissues and the serum. Two key angiogenic factors, which include vascular endothelial growth factor-A (VEGF-A) and angiopoietin-2 (Ang-2), are controlled by HIF-1α. Ang-2 promotes vasculature remodeling by inhibiting the associated angiopoietin-1 protein (24). Angiogenesis is also crucial for tumor progression and development. Administering VEGF-specific monoclonal antibodies to mice suppressed tumor development (25). VEGF-A also mediates vascular permeability, which is linked with malignant effusions (26). Furthermore, neo-angiogenesis and VEGF expression in NSCLC are markers of a poor prognosis (27). Surprisingly, VEGF receptor 2 (VEGFR2), VEGF-A and C, matrix metalloproteinase-2 (MMP-2), and MMP-9 were overexpressed in the lungs and tumors of hypoxic mice. VEGF-A and MMP-9 also increase in hypoxic malignancies (28). Angiogenesis is controlled by tie receptors, which are involved in vascular homeostasis, and VEGF receptors are the essential components of angiogenesis. VEGF receptor 1 (VEGFR1) and Tie2 are downregulated in hypoxic mouse tumors, which may cause aberrant vascular leakage linked to tumor invasion (28). In the 3-methylcholanthrene/butylated hydroxytoluene animal model, which is an inflammation-dependent priming-promoting model, chronic persistent alveolar hypoxia increased the expression of epidermal growth factor receptor (EGFR), fibroblast growth factor receptor 2, and platelet-derived growth factor receptor, which are known to promote tumor growth and angiogenesis. However, intermittent hypoxia did not induce the same increase in these growth factor receptors (28).

In addition to VEGF family, the downstream target genes of HIF, which include nuclear factor kappa B (NF-κB) and toll-like receptors, link hypoxia to inflammation (23). Inflammation significantly contributes to an increased susceptibility to lung cancer in patients with COPD. In addition, several NF-κB (key regulators of inflammation) target genes involved in carcinogenesis, including interleukin-6, MMP-9, and cyclooxygenase 2, are also downstream genes of HIF-1α (29). Hypoxia induces NF-κB expression (30). Furthermore, NF-κB stimulates cell proliferation, inhibits programmed cell death, facilitates tumor dissemination, and modifies tumor metabolism in the context of chronic inflammation (29). Moreover, NF-κB can also decrease p53 stability to promote carcinogenesis and can collaborate with HIF-1α to enable the activation of tumor-promoting gene promoters (31). NF-κB also directly regulates the expression of genes encoding MMPs such as MMP-9, leading to extracellular matrix remodeling, which promotes cancer cell distribution in the vicinity (29).

**Figure 1:**
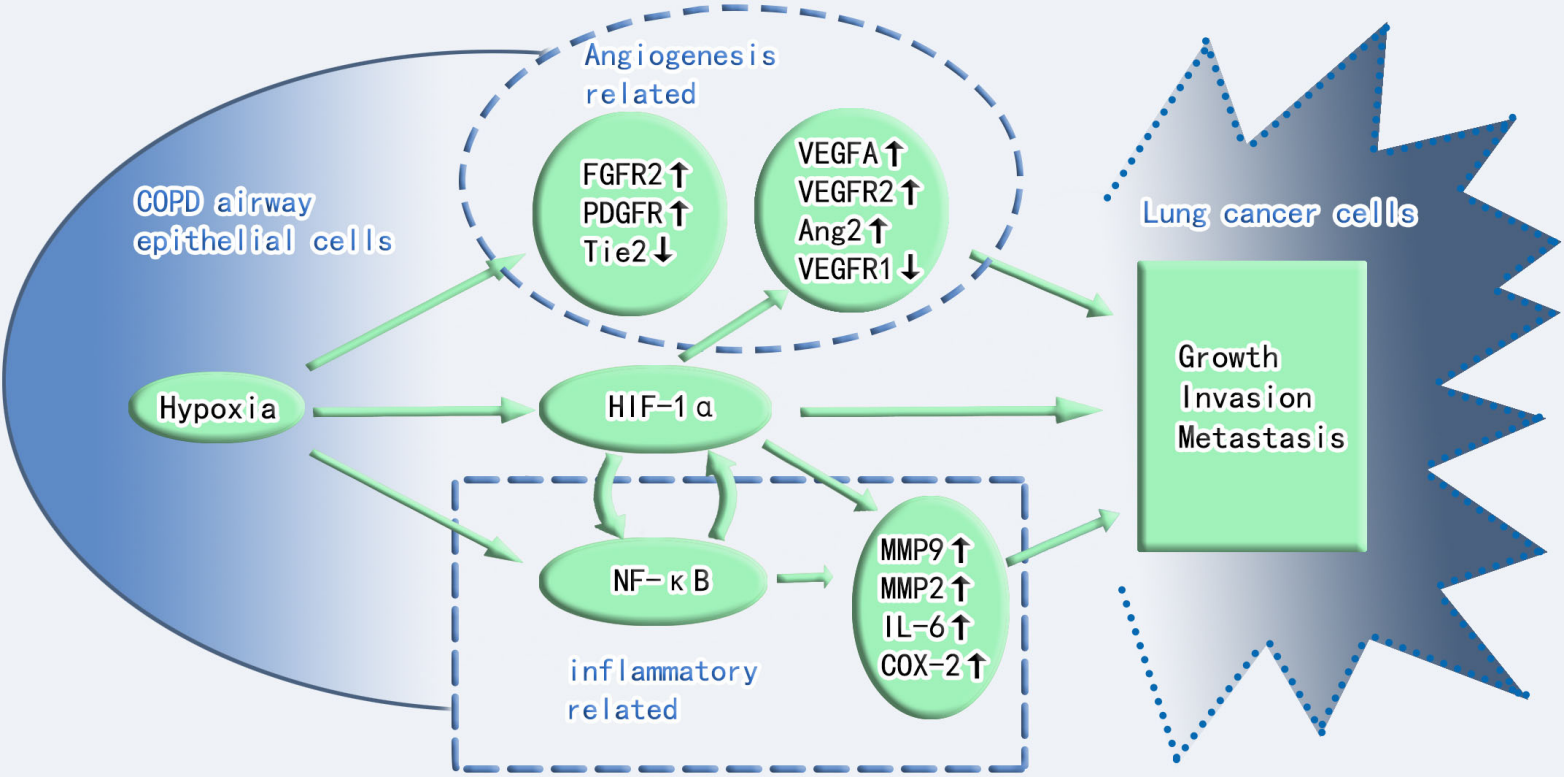
Crosstalk between hypoxia and inflammation in COPD and lung cancer toxicity. Angiogenesis, HIF-1α, and inflammation play important roles in the progression of COPD to lung cancer. Angiogenesis and inflammation cross-talk through HIF-1α. FGFR2, fibroblast growth factor receptor 2; PDGFR, platelet-derived growth factor receptor; Tie2, Tie2 (a member of the receptor tyrosine kinase family and the receptor for angiopoietin); VEGF-A, vascular endothelial growth factor-A; VEGFR2, vascular endothelial growth factor receptor 2; VEGFR1, vascular endothelial growth factor receptor 1; Ang2, angiopoietin-2; NF-κB, nuclear factor kappa B; IL-6, interleukin-6; COX-2, cyclooxygenase 2; MMP9, matrix metalloproteinase-9; MMP2, matrix metalloproteinase-2.

Furthermore, chronically inflamed tissue leads to a hypoxic environment that prevents hypoxia-inducible factor prolyl hydroxylase (PHD), thereby regulating HIF stability. Interleukin-1β and lipopolysaccharide are proinflammatory cytokines that increase the basal transcription rate of HIF messenger RNA probably because NF-κB binds to the HIF-1α promoter and upregulates HIF-1α (32). NF-κB also promotes HIF-1α activation and enhances HIF-1α expression during hypoxia (33). In contrast, PHD and HIF-1α inhibitors (FIH) regulate NF-κB activation by controlling inhibitor of kappa B kinase (IKKβ)complex activity, which is a regulatory component of NF-κB (34) (**Table 1** and **Figure 1**).

**Table 1:** Hypoxia and inflammation combined in cancer.

Cytokines	Induction method	Expression in hypoxic tumor	Role of cytokines in hypoxic tumors after being modulated
**HIF-1α**	Induced by hypoxia	Up	Promote angiogenesis and tumor growth (24, 31) Promote inflammation and a variety of inflammatory mediators (29–31)
**PDGFR**	Induced by hypoxia	Up	Promote angiogenesis and tumor growth (28)
**FGFR2**	Induced by hypoxia	Up	Promote angiogenesis and tumor growth (28)
**Tie 2**	Induced by hypoxia	Down	Cause aberrant vascular leakage associated with tumor invasion (28)
**NF-κB**	Co-induced by hypoxia and inflammation	Up	Promote inflammation (29, 31) Stimulate cell proliferation (29) Inhibit programmed cell death (29) Facilitate tumor dissemination (29) Promoting carcinogenesis (31) Facilitate the activation of tumor-promoting gene promoters (30, 31)
**VEGF family** **VEGF-A** **VEGFR1** **VEGFR2**	Target of HIF-1α	Up Down Up	Promote angiogenesis and tumor growth (26, 29) Mediate vascular permeability associated with malignant effusions (27) Cause aberrant vascular leakage associated with tumor invasion (29) Promote tumor growth (29)
**Ang-2**	Target of HIF-1α	Up	Promote remodeling of the vasculature (22)
**MMP-9**	Common target of HIF-1α and NF-κB	Up	Promote inflammation (30, 32) Promote the spread of cancer cells in the vicinity (30) Promote tumor development (32)
**MMP-2**	Common genes of HIF-1α and NF-κB	Up	Promote inflammation and VEGF release from the extracellular matrix (30) Promote tumor invasion (32)
**IL-6**	Common genes of HIF-1α and NF-κB	Up	Promote inflammation and tumor development (29, 31)
**COX2**	Common genes of HIF-1α and NF-κB	Up	Promote inflammation and tumor development (30, 32)

PDGFR, platelet-derived growth factor receptor; FGFR2, fibroblast growth factor receptor 2; NF-κB, nuclear factor kappa B; VEGFR2, vascular endothelial growth factor receptor 2; Ang-2, angiopoietin-2; IL-6, interleukin-6; COX-2, cyclooxygenase 2.

## HIF-1

### HIF-1 initiation

HIF-1 is essential for the body’s adaptive regulatory response to changes in the oxygen environment and is involved in several physiological and pathological processes in the body, as well as in the origin of many disorders. HIF-1 is a heterodimeric transcription factor with three oxygen-sensitive alpha subunits, HIF-1α, HIF-2α, and HIF-3α, and one constitutive beta subunit, HIF-1β (35). Interestingly, studies on the mammalian HIF-1α have significantly contributed to the knowledge of the structure and regulation of transcription factors. Both HIF-1α and HIF-1β possess a basic helix loop helix (bHLH) domain and two Per-Arnt-Sim (PAS) domains, which include PAS-A and PAS-B (36). Additionally, the bHLH and PAS structural domains mediate the heterodimerization of HIF-1α and HIF-1β. A basal region that precedes the N-terminus of the bHLH domain binds this heterodimer to the hypoxia response element (HRE)-DNA motifs of the target gene promoters (36). The two transactivation domains (TADs) are categorized as follows: NH_2_-terminal TAD (N-TAD) and COOH-terminal TAD (C-TAD), which are located at the NH_2_-and COOH terminuses, respectively. N-TAD is found within the oxygen-dependent degradation domain (ODDD), which is constrained to amino acid residues (400–600) of HIF-1α. Conversely, ODDD and C-TAD control the activity and stability of the α-subunit, which will be described in detail below. Furthermore, the amino acid sequence (576–785) of the inhibitory domain (ID) that prevents the transcriptional activity of N-TAD and C-TAD under normoxic conditions separates N-TAD and C-TAD (36). Summarily, HIF-1 signaling begins with its dimerization. HIF-1α migrates to the nucleus and dimerizes with HIF-1β. Subsequently, the dimer binds to the promoters of target genes containing HREs, initiating the transcription of multiple genes, including *EGFR* (35), which is involved in cellular adaptation to hypoxia, metabolism, and cellular function (37). Furthermore, the ability of HIF-1 to cause hypoxia-induced transactivation significantly depends on its HIF-1α subunit (36) (**Table 2**).

**Table 2:** The Location of the HIF-1 Partial Functional Domains/Regions.

Feature key	Position(s)	Description	Graphical view	Length
Domain	17–70	bHLH	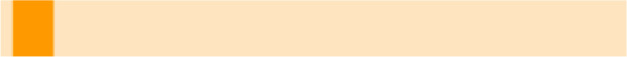	54
Domain	85–158	PAS 1	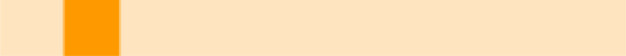	74
Domain	228–298	PAS 2	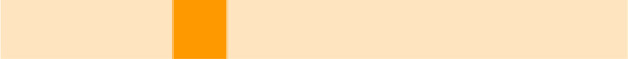	71
Domain	302–345	PAC	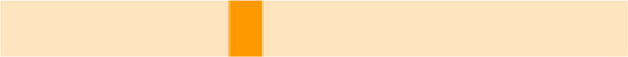	44
Region	1–401	Interaction with TSGA10	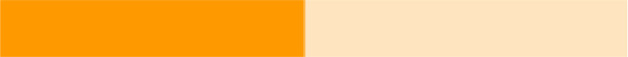	401
Region	1–30	Disordered	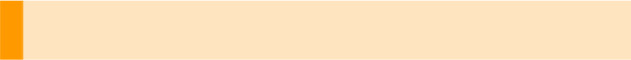	30
Region	21–30	DNA-binding	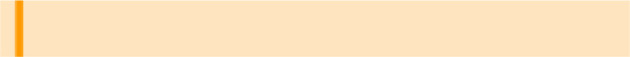	10
Region	170–191	Required for heterodimer formation with ARNT	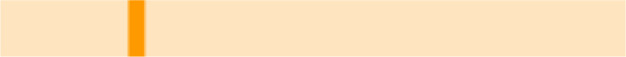	22
Region	380–417	N-terminal VHL recognition site	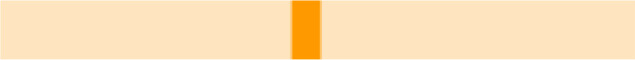	38
Region	401–603	ODDD	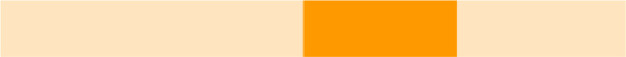	203
Region	494–520	Disordered	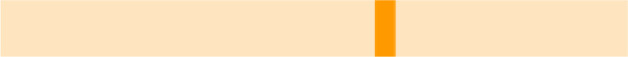	27
Region	531–575	N-TAD	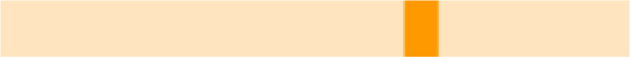	45
Region	556–572	C-terminal VHL recognition site	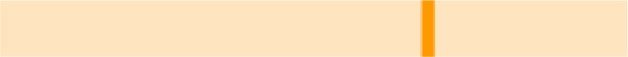	17
Region	576–785	ID	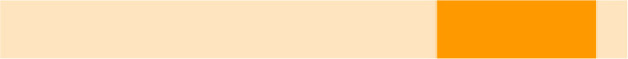	210
Region	642–688	Disordered	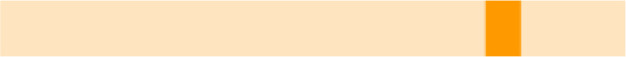	47
Region	786–826	C-TAD	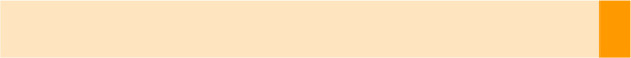	41

HIF-1, hypoxia-inducible factor 1; bHLH, basic helix loop helix; PAS 1, Per-Arnt-Sim 1; PAS 2, Per-Arnt-Sim 2; PAC, PAC motifs occur C-terminal to a subset of all known PAS motifs; TSGA10, testis-specific gene 10 (a protein-coding gene); ARNT, aryl hydrocarbon receptor nuclear translocator; VHL, von Hippel–Lindau protein; ODDD, oxygen-dependent degradation domain; N-TAD, NH_2_-terminal transactivation domain; ID, inhibitory domain; CTAD, COOH-terminal transactivation domains.

### Hydroxylation regulates HIF-1α

**Figure 2:**
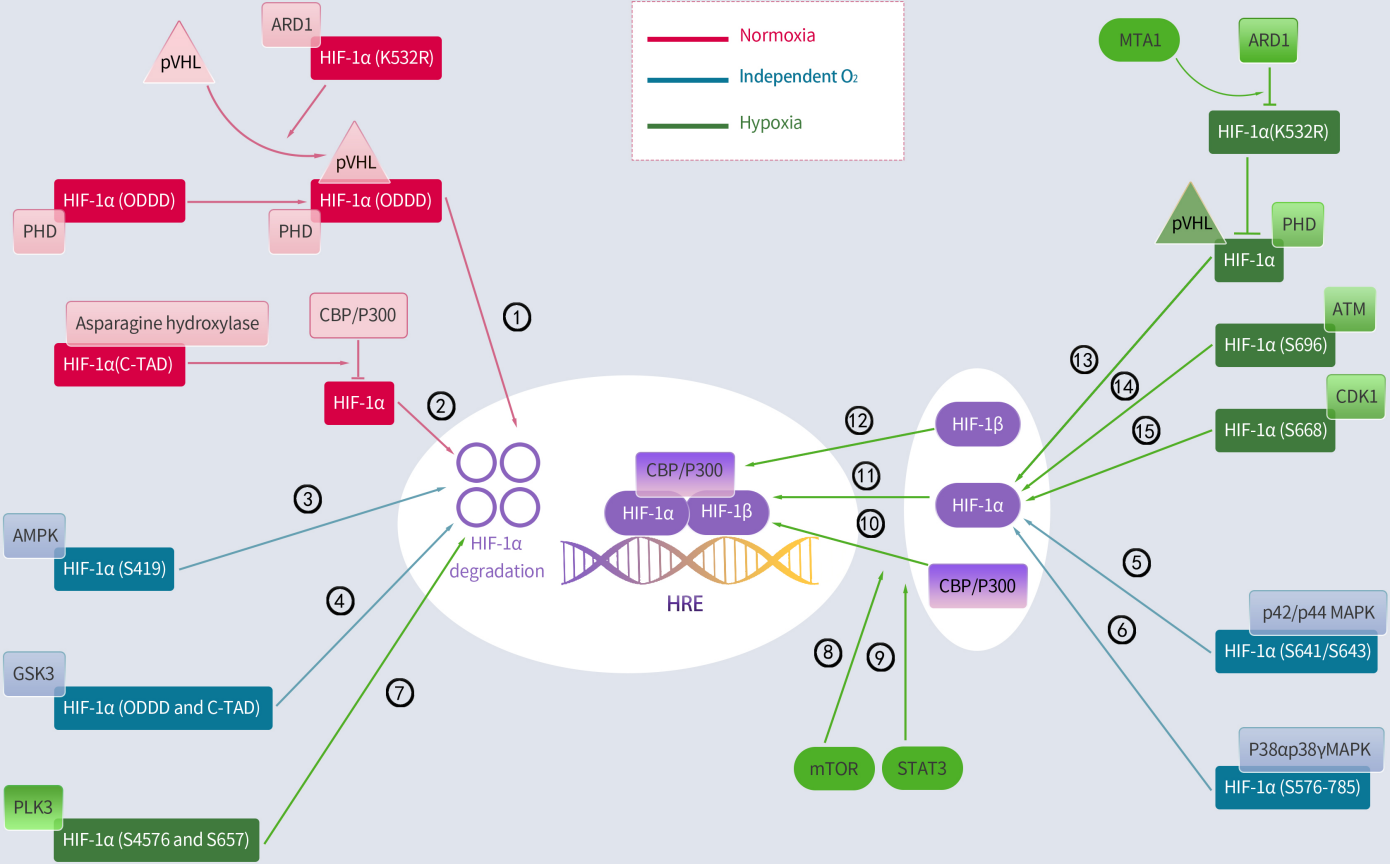
Regulation of HIF-1α by hydroxylation, phosphorylation and acetylation. Activated regulatory pathways under normoxia: ①②; Activation of the following pathway is less associated with oxygen: ③④⑤⑥;Activated regulatory pathways under hypoxia: ⑦⑧⑨⑩⑪⑫⑬⑭⑮. PHD, prolyl hydroxylase; pVHL, Von Hippel-Lindau protein; ARD1, arrest defective-1; CBP/P300, cAMP response element binding protein (CREB)-binding protein and P300; ODDD, oxygen-dependent degradation domain; AMPK, adenosine 5’-monophosphate (AMP)-activated protein kinase; GSK3, glycogen synthase kinase-3; PLK3, polo-like kinase-3; MTA1, metastasis-associated protein 1; ATM, ataxia and telangiectasia mutated; p42/p44 MAPK, mitogen-activated protein kinase; CDK1, cyclin-dependent kinase-1; mTOR, mammalian target of rapamycin; STAT3, signal transducer and activator of transcription 3.

Several environmental factors influence HIF-1 expression. Interestingly, the principal mechanism by which HIF-1 is regulated is hydroxylation, with most of the mechanism occurring in the α-subunit. In resting cells, PHD, which includes PHD1, PHD2, and PHD3, hydroxylates proline residues within the ODDD region of HIF-1α, enabling the ubiquitination of HIF-1α by the von Hippel–Lindau protein (pVHL) E3 ubiquitin ligase (36, 38). Therefore, the PHD and pVHL proteins primarily facilitate HIF-1α degradation. Additionally, since PHD depends on oxygen, the baseline level of HIF-1α remains low under normoxic conditions with a half-life of less than 5 minutes (39). Conversely, hypoxia inhibits PHD and reduces HIF-1 hydroxylation, resulting in HIF-1 accumulation, which translocates to the nucleus and binds to its coactivator (38). Moreover, asparagine hydroxylase, under normal conditions, hydroxylates asparagine residues 803 and 847 within the HIF-1α C-TADs. It prevents HIF-1α from interacting with its coactivator, which is a cyclic adenosine monophosphate response element binding protein-binding Protein/P300 (CBP/p300) (36, 40). In contrast, this FIH function is suppressed under hypoxic conditions, which enables the binding of HIF-1α to its coactivators and boosts the expression of its downstream target genes. Additionally, the signal transducer and activator of transcription 3 enhance HIF-1α activity by blocking VHL from binding to HIF-1α and facilitating the coactivator CBP/p300 to bind to HIF-1α. Additionally, the mammalian target of rapamycin (mTOR) induces HIF-1α binding to its coactivator (41) (**Figure 2**).

### Acetylation regulates HIF-1α

Moreover, lysine acetylation can alter the HIF-1α protein at various sites, resulting in obvious downstream effects. However, these findings remain controversial. A report indicated that the lysine (K532) residue of HIF-1α directly interacts with arrest defective-1 (ARD1: a protein acetyltransferase) to acetylate HIF-1α, thereby enabling HIF-1α binding to pVHL and finally HIF-1α degradation. This study further proved that pVHL-dependent HIF-1α degradation is associated with intra-ODDD acetylation. Metastasis-associated protein 1 (MTA1) is a member of the MTA family that opposes the acetylation function of ARD1 and upregulates the expression of histone deacetylase 1, which induces HIF-1α deacetylation at the K532R site, thereby improving the transcriptional activity and stability of the HIF-1α protein (36). A hypoxic environment notably enhances MTA1 expression (42). However, a report indicates that ARD1 neither acetylates nor destabilizes HIF-1α (43). Similarly, ARD1 inhibition and overexpression had minimal effect on basal HIF-1α levels or hypoxic responses (44). Additionally, the CBP/p300-associated factor acetylates the lysine residue (K674), which elevates HIF-1α levels and promotes HIF-1α binding to P300 (45). In particular, Geng et al. (46) demonstrated that P300 stabilizes HIF-1α by acetylating lysine residues (K709). Summarily, most current studies indicate that HIF-1α is acetylated and deacetylated, leading to its removal and accumulation, respectively (**Figure 2**).

### Phosphorylation regulates HIF-1α

Protein kinases control HIF-1α activity and phosphorylate specific HIF-1α residues under normoxic conditions. Cyclin-dependent kinase-1 (CDK1) phosphorylates HIF-1α at serine 668, increasing the expression of HIF-1α and its target genes and boosting tumor angiogenesis, proliferation, and growth (47). Similarly, ataxia and telangiectasia mutated (ATM) proteins phosphorylate the inhibitory domain residue S696 to stabilize HIF-1α (48). Conversely, polo-like kinase-3 (P3) phosphorylates residues S576 and S657 to destabilize HIF-1α (49). Moreover, glycogen synthase kinase-3 (GSK3) also phosphorylates residues S551, T555, S589, T498 S502, S505, T506, and S510 in ODDD and N-TAD, which enhances HIF-1α degradation (50–52). In the human liver hepatocellular cell lines (HepG2), chronic hypoxia induced an increase in GSK3 activity, which decreased HIF-1α protein levels (52); however, this effect was undetected in other cell types. Additionally, adenosine 5’-monophosphate-activated protein kinase (AMPK) phosphorylates S419 in HIF-1α to downregulate HIF-1α (53). Additionally, two members of the p38 mitogen-activated protein kinase (MAPK) family, p38α and p38γ, can phosphorylate the amino acid 576–785 domain of HIF-1α, thereby contributing to its stability (54). The activation of the p42/p44 MAPK pathway induces HIF-1α by phosphorylating S641/S643 in HIF-1α (55–57).This study further found that p42/p44 MAPK activation was sufficient to promote the transcriptional activity of HIF-1 in the VEGF promoter mutated at a MAPK-sensitive site (SP1/AP2-88-66 site) (57) (**Figure 2**).

## COPD and lung cancer exhibit aberrant HIF-1α expression

### HIF-1α corresponds with COPD severity

COPD causes a hypoxic environment for HIF-1α. Numerous studies have proven that HIF-1α is overexpressed in the lungs of patients (58–60). Wang et al. (59) measured HIF-1α serum levels using enzyme-linked immunosorbent assay. They discovered that serum HIF-1α levels were higher in stable patients with COPD than in the general population and that these higher serum levels were positively correlated with the Global Initiative for Chronic Obstructive Lung Disease (GOLD) classification, the United Kingdom modified Medical Research Council (mMRC) score, and medical history. Therefore, this result suggests that increased HIF-1α serum levels are associated with COPD progression. Additionally, Fu et al. (61) assessed HIF-1α expression in the lung tissues of 102 smokers with or without COPD. Patients with COPD had increased levels of HIF-1α and its downstream target genes, including VEGF and VEGFR2, in their lung tissue compared with healthy individuals and nonsmokers without COPD. Furthermore, COPD severity has been associated with HIF-1α expression and downstream target genes. In another study, Zhang et al. (60) investigated the bronchoalveolar lavage fluid from patients with COPD and healthy participants. They found that HIF-1α was overexpressed, and the production of inflammatory markers was elevated in patients with COPD through the activation of the EGFR/PI3K/AKT pathway. Moreover, in a feedback loop, the lung inflammation-induced EGFR/PI3K/AKT pathway upregulates HIF-1α expression, eventually exacerbating COPD.

### HIF-1α stimulates tumor development

Increased levels of HIF-1α expression promote tumor growth, whereas a reduction in its activity inhibits tumor growth (62). HIF-1α levels in lung cancer tissue samples are excessively high and play a crucial role in tumor genesis, development, and metastasis (63–65). Infantino et al. (66) demonstrated that HIF-1α contributes to the regulation of glutamine and serine metabolism as well as the one-carbon cycle and fatty acid metabolism in hypoxic cancer cells, thereby reprogramming cancer cell metabolism under hypoxic conditions. Additionally, these changes promote cell survival, proliferation, and tumor growth. Furthermore, HIF-1α overexpression enhanced tumor development, vascularization, and energy metabolism, whereas a decrease in its activity had the opposite impact during *in vitro* xenograft tests (67–70). Moreover, cigarette smoke extract, which is a prevalent risk factor for COPD and lung cancer, also elevates HIF-1α in a concentration-dependent manner (71). In addition, VEGFR, which is one of the essential target genes downstream of HIF-1α, adapts cancer cells to the hypoxic environment and stimulates the formation of new blood vessels in the tumor, provides nutrients and oxygen to promote tumor growth and metabolism, and presents a major pathway for cancer cells to metastasize to distant organs (72).

### HIF-1α links COPD with lung cancer

HIF-1α may increase the risk of cancer in patients with COPD. Although when the gene encoding HIF-1α was disrupted in the lung epithelium of a K-ras mutant mouse model (CC-LR), independent of COPD-like airway inflammation, the number of surface tumors in the mouse lung, tumor angiogenesis, and tumor cell proliferation were considerably reduced (73). However, COPD- and adenocarcinoma-like phenotypes were observed in their offspring when CC-LR mice were bred with transgenic animals overexpressing human HIF-1α in airway epithelial cells. According to the same study, the CC-LR mice with overexpressed HIF-1α in the COPD airway epithelium developed substantial emphysema. Additionally, they possessed an aggressive metastatic phenotype, with increased tumorigenesis, angiogenesis, and cell proliferation. Polosukhin et al. (74) analyzed 55 bronchial biopsies from smokers with COPD and found that HIF-1α contributes to the progression of precancerous epithelial lesions in the airways of smokers with chronic airway inflammation. Furthermore, HIF-1α promotes cigarette smoke exposure-induced malignant transformation of bronchial epithelial cells, which is a process dependent on AKT/NF-κB pathway (75). Moreover, HIF-1α also encourages epithelial-mesenchymal transition and the acquisition of cancer stem cell-like characteristics in the bronchial epithelium (76, 77). Therefore, these findings indicated a link between COPD-associated airway inflammation, HIF-1α, and lung cancer.

## Targeting HIF-1α in lung cancer

The mechanism of action of HIF-1α in lung cancer, HIF-1α targets, and HIF-1α pharmacology are of significant interest to researchers. In NSCLC experiments, several substances, such as the HIF-1α inhibitor LW6 (78), chetomin (79), gamma-linolenic acid (80), propofol (81), a novel mycotoxin-derived compound GL331 (82), resveratrol (83), sevoflurane (84), flavanols (85), MiR-199a (86, 87), and connective tissue growth factor (88), possess anticancer properties focusing on cancer cell proliferation and invasion capabilities, by downregulating HIF-1α levels. Interestingly, digoxin inhibits hypoxia-induced VEGF, HIF-1α, and N-myc downstream-regulated genes 1 (NDRG1) in a concentration-dependent manner in A549 cells (89). Topotecan and etoposide inhibited the hypoxia-induced expression of HIF-1α protein in NSCLC cell lines in a dose- and time-dependent manner (90). Conversely, the combination of emodin and cisplatin downregulated the multidrug resistance-1 gene and HIF-1α production in lung tumor cells (91), which restricted cancer cell proliferation, adhesion, migration, and tumor angiogenesis. Additionally, PX-478, which is a potent small-molecule HIF-1α inhibitor, had a substantial anticancer effect in two adenocarcinoma models, PC14-PE6 and NCI-H441, and two small cell lung cancer (SCLC) models, NCI-H187 and NCI-N41 (92). Furthermore, cytokine signaling 3 (SOCS3) suppresses HIF-1α expression, reducing the progression and spread of SCLC (93). Sulforaphane inhibits HIF-1α expression under hypoxic conditions probably because of its H_2_S-donating characteristics (94, 95). Sphingosine kinase 1 (96) and andrographolide (97) prevent HIF-1α expression in NSCLC cells, although no link with anticancer activity was detected. Moreover, SU5416 and KRN633 are VEGFR tyrosine kinase inhibitors that reduce HIF-1α expression in various cancer cells (excluding lung cancer) (98). Therefore, future studies are necessary to determine whether they have similar effects on lung cancer cells (**Table 3**).

**Table 3:** Substances that can target HIF-1α in lung cancer.

Medicine/Protein	Experimental cells	*In vivo/vitro*	Impact on HIF-1α	Ways to influence HIF-1α	Experimental results
LW6	A549 cells	*In vitro*	Inhibit levels of HIF-1α	Upregulate pVHL	Induced apoptosis
Digoxin	A549 cells	*In vitro*	Inhibit levels of HIF-1α	Suppress the expression of VEGF, NDRG1, and HIF-1α	Suppressed growth of cells
Chetomin	H1299 and H460 cells	In mice and vitro	Inhibit levels of HIF-1α	Block p300-HIF-1α interaction	Antitumor activity
SOCS3	NCI-H446 cells	In mice and vitro	Inhibit levels of HIF-1α	Downregulate the activation of AKT	Inhibited the proliferation and angiogenesis of cells
GLA	Calu-1 and SK-MES-1 cells	*In vitro*	Inhibit levels of HIF-1α	Suppress the HIF1α-VEGF pathway	Inhibited the proliferation and invasion of cells
GL331	CL1-5 cells	*In vitro*	Inhibit levels of HIF-1α and VEGF	Decrease the binding of CL1-5-derived nuclear components to the promoter of HIF-1alpha gene	Suppressed the angiogenesis of cells
Propofol	A549 cells	*In vitro*	Inhibit levels of HIF-1α	/	Suppressed the migration and invasion of cells
Resveratrol	LLC cells	In mice	Inhibit levels of HIF-1α	/	Antitumor activity
Sevoflurane	A549 cells	*In vitro*	Inhibit levels of HIF-1α	/	Suppressed the growth and metastasis of cells
Flavanols	A549 cells	In mice and vitro	Inhibit levels of HIF-1α and VEGF	/	Antitumor and anti-angiogenic activity;
CTGF	CL1-5 and A549 cells	In mice	Inhibit levels of HIF-1α	/	Suppressed the proliferation, migration, invasion, and angiogenesis of cells
Top I and Top II inhibitor	A549, H460, H1299, and H358 cells	*In vitro*	Inhibit levels of HIF-1α	/	Seemed useful for overcoming the therapeutic resistance induced by tumor hypoxia in NSCLC
MiR-199a	NCI-H520 cells	In mice	Inhibit levels of HIF-1α and VEGF	/	Suppressed the proliferation of cells
PX-478	PC14-PE6, NCI-H441, NCI-H187 and NCI-N417 cells	In mice	Inhibit levels of HIF-1α	/	Antitumor activity
Combination of emodin and cisplatin	A549 and cells	*In vitro*	Inhibit levels of HIF-1α	/	Antitumor activity
Sphingosine kinase 1	A549 cells	*In vitro*	Inhibit levels of HIF-1α	/	/
Andrographolide	A549 cells	*In vitro*	Inhibit levels of HIF-1α	Increase PHD2 and decrease VEGF	/
SU5416 and KRN633	HeLa, A431, MCF7 HCT 15 and HCT 116 cells	*In vitro*	Inhibit levels of HIF-1α	Inhibit both AKT and ERK phosphorylation signaling pathways	/

Experimental cell types: (a) human lung adenocarcinoma cells: A549, CL1-5, LLC, PC14-PE6, and NCI-H441; (b) human NSCLC cells: H1299, H460, H358, NCI-H520, and SK-MES-1; (c) human SCLC cells: NCI-H446, NCI-H187, and NCI-N417; (d) human lung cancer cells: Calu-1; (e) other cancer cells: human cervical carcinoma cell line, HeLa; human epithelial carcinoma cell line, A431; human breast carcinoma cell line, MCF7; human colorectal carcinoma cell lines, HCT 15 and HCT 116.

Topoisomerase (Top) I inhibitor, topotecan; Top II inhibitor, etoposide; HIF-1α, hypoxia-inducible factor-1 alpha; VEGF, vascular endothelial growth factor; PHD2, prolyl hydroxylase 2; ERK, extracellular regulated kinase; NSCLC, non-small cell lung cancer; AKT, protein kinase B; NDRG1, N-myc downstream-regulated genes 1; pVHL, von Hippel–Lindau protein.

## Targeting HIF-1α could reduce COPD-related oncogenic effects

The above evidence implies that an increase in HIF-1α levels in COPD leads to the progression of COPD to lung cancer progression. Conversely, inhibiting HIF-1α expression in COPD may delay or block this process. However, some studies have proven that HIF-1α plays a crucial role in the energy metabolism of inflammatory cells and that preventing it causes severe immunodeficiency (24). In a preliminary assessment of HIF interference in inflammatory diseases, the hydroxylase inhibitors dimethyloxalylglycine and FG4497 had a significant protective effect on two models of inflammatory bowel disease (IBD), such as dextran sodium sulfate and 2,4,6-trinitrobenzene sulfonic acid (99, 100). Notably, the respiratory and digestive systems have a common embryonic origin in the primitive foregut. Consequently, their structural similarities may explain their comparable immunological responses to noxious stimuli (101). Furthermore, the closely related IBD and COPD are chronic inflammatory diseases of the digestive and respiratory systems, respectively. Moreover, exposure to cigarette smoke is also a common risk factor for both diseases. Accordingly, both disease states show increased levels of innate lymphocytes and unconventional T cells, such as γδT cells, and myeloid cells, including neutrophils, eosinophils, and macrophages (102). Because VEGF is an important downstream target gene of HIF, we also considered whether blocking its pathway would have a similar effect, and we searched for relevant information. Although evidence that VEGF inhibitors can improve COPD exists, whether they can delay or block the progression of COPD to lung cancer has not been elucidated. Therefore, additional studies are required to clarify this issue. Accordingly, we briefly describe the drugs that can target HIF-1α at the level of COPD, as well as the therapies that can accomplish this effect.

### Non-coding RNA

Non-coding RNAs (ncRNAs) are RNAs that do not code for proteins and are categorized into short microRNAs (miRNAs) and long ncRNAs. Targeting ncRNAs is a promising therapeutic strategy since they are involved in various complex pathophysiological processes, such as immune system development and function, immunological diseases, and neurodevelopmental and neurological disorders (103).

Interestingly, miRNAs are prevalent in most somatic tissues. Additionally, they are generally 22 nucleotides long and created by two ribonuclease III proteins, Drosha and Dicer (104), which are essential for gene expression regulation (105), predominantly through translational repression or messenger RNA degradation (106). Additionally, miRNAs control the expression of HIF-1α (107, 108). Notably, Shiro et al. (109) were the first to reveal that individuals with COPD/emphysema had higher lung tissue expression of miR-34a and miR-199-5p. Specifically, they observed that human pulmonary microvascular endothelial cells (HPMVECs) transfected with the miR-199a-5p gene showed a reduced HIF-1α protein expression. Additionally, miR-199-5p expression was elevated in HPMVECs transfected with miR-34a precursor gene. In a recent study, Wu et al. (110) found that miR-125a-5p levels were higher in normal smokers and COPD smokers than in the healthy population. Furthermore, researchers have used smoke to induce COPD in mouse models. They found that miR-125a-5p targeted the 3’-untranslated region of Sp1 mRNA. Sp1 promotes sirtuin 1 (SIRT1) expression, and SIRT1 deacetylates HIF-1α, thereby downregulating HIF-1α on Lys674. Therefore, when miR-125a-5p was knocked down, HIF-1α expression was increased. Similarly, Li et al. (105) conducted a cell culture experiment with lung fibroblasts (MRC-5) and found that miR-186 decreased HIF-1α by directly targeting HIF-1α mRNA. These results indicate that the increased expression of miR-199a-5p, miR-34a, miRNA-186, or miR-125a-5p in the lung tissue of patients with COPD may inhibit HIF-1α production, consequently reducing COPD carcinogenesis. Furthermore, numerous miRNA-targeted therapies, including those for solid tumors, viral hepatitis C, atherosclerosis, myocardial infarction, renal fibrosis, diabetes and its complications, and COPD, have been employed in animal trials and perhaps attained clinical development (111). Despite insufficient studies on the application of miRNAs to lower HIF-1α expression in patients with COPD, this treatment strategy appears beneficial and promising.

### Sodium hydrosulfide

Sodium hydrosulfide (NaHS) is an H_2_S donor. Wu et al. (112) discovered that NaHS reversed HIF-1α accumulation during hypoxia and that this effect persisted even after the degradation of the ubiquitin-proteasome pathway was inhibited. Guan et al. (113) also discovered that NaHS significantly reduced cigarette smoke-induced HIF-1α protein expression and enhanced PHD2 protein levels in a mouse model and in *in vitro* A549 alveolar epithelial cells. Notably, NaHS reduces inflammation, epithelial cell damage, and apoptosis by blocking HIF-1α signaling. These findings suggest that NaHS may be a novel therapy for COPD. Additionally, NaHS protects against stress-induced lung injury because of its antioxidative, anti-inflammatory, antifibrotic, and antiapoptotic properties (114). Over the past few decades, several H_2_S-based treatment approaches, including those based on H_2_S donation and the restriction of H_2_S generation, have been identified. H_2_S donors can be obtained through the inhalation of H_2_S gas, parenteral or enteral injection of H_2_S salts, or other methods. Furthermore, H_2_S donors can be rapidly or slowly released, such as NaHS, Na_2_S, or GY4137, which is a prototype chemical employed in several *in vivo* and *in vitro* studies (115). These findings indicate that NaHS may be used as a therapy to prevent the progression of COPD to lung cancer.

### Pyrrolidine dithiocarbamate

Pyrrolidine dithiocarbamate (PDTC) is an antioxidant that is believed to be a potent NF-κB inhibitor (116). Briefly, rats were orally administered PDTC, treated by the intratracheal instillation of lipopolysaccharide, and subsequently exposed to cigarette smoke, as described by Jiang et al. (117). They found a decreased HIF-1α expression through NF-κB inhibition in the COPD group caused by PDTC pretreatment. Therefore, PDTC may be used to lower HIF-1α expression in patients in the future. Moreover, PDTC, which is an NF-κB inhibitor, may also be effective for treating COPD.

### *Lycium barbarum* polysaccharides

*Lycium barbarum* polysaccharides (LBP), comprising glucose, arabinose, galactose, mannose, rhamnose, and xylose, are the primary bioactive components of the Chinese herbal remedy, *Lycium barbarum*. Interestingly, multiple conditions, such as cancer, aging, fatigue, colitis, stroke, diabetes, Alzheimer’s disease, and glaucoma, have been proven to benefit from LBP (118). Chen et al. (119) demonstrated that oral LBP (5 mg/mL, 100 mL twice daily for 2 weeks) improved COPD symptoms and significantly decreased blood HIF-1α levels. In this study, oral LBP elicited moderate adverse effects, including constipation, dry mouth, inflamed gums, and gastrointestinal reactions. However, these unfavorable reactions to LBP diminished when the patient stopped taking LBP, signifying that the side effects associated with LBP were transient and reversible. Furthermore, animal studies reveal that LBP has no negative or teratogenic effects on sperm (120), although further studies are warranted to validate this claim. LBP may benefit patients with COPD and reduce lung cancer incidence; however, this also requires additional clinical verification.

## Conclusion

HIF-1α signaling plays a key role in the progression of COPD to lung cancer and is a potential therapeutic target to delay the progression. Additional insight into the processes that govern HIF-1α expression and activation during COPD inflammatory carcinogenesis would be advantageous in developing COPD-specific HIF-1α therapies.

## Author contributions

The structure of the manuscript was decided on by all the authors. Y-RX wrote the first draft of this manuscript. A-LW worked on the figures. All authors contributed to the article and approved the submitted version.

## Funding

This work was supported by grants from the National Natural Science Foundation of China (Nos. 81870028 and 81470241) and the Zhejiang Provincial Program for the Cultivation of High-level Innovative Health Talents (No. A-2017-CXCR02).

## Acknowledgments

The authors thank Yaqing Li for editing the manuscript.

## Conflict of interest

The authors declare that the research was conducted in the absence of any commercial or financial relationships that could be construed as potential conflicts of interest.

## Publisher's note

All claims expressed in this article are solely those of the authors and do not necessarily represent those of their affiliated organizations, or those of the publisher, the editors and the reviewers. Any product that may be evaluated in this article, or claim that may be made by its manufacturer, is not guaranteed or endorsed by the publisher.

## Glossary

**Table:**

COPD	chronic obstructive pulmonary disease
HIF-1	hypoxia-inducible factor 1
HIF-1α	hypoxia-inducible factor 1alpha
PD-L1	programmed cell death 1 ligand 1
CT	computed tomography
NSCLC	non-small cell lung cancer
VEGF-A	vascular endothelial growth factor-A
Ang-2	angiopoietin-2
MMP-2	matrix metalloproteinase-2
VEGFR2	VEGF receptor 2
VEGFR1	VEGF receptor 2
EGFR	epidermal growth factor receptor
NF-κB	nuclear factor kappa-B
PHD	prolyl hydroxylase
FIH	HIF-1α inhibitor
IKKβ	inhibitor of kappa B kinase
bHLH	basic Helix Loop Helix
PAS	Per-Arnt-Sim
HRE	hypoxia response element
TADs	trans-activating structural domains
N-TAD	NH2-terminal TAD
C-TAD	COOH-terminal TAD
ODDD	oxygen-dependent degradation domain
ID	inhibitory domain
pVHL	von Hippel-Lindau protein
CBP/P300	cyclic adenosine monophosphate response element binding protein-binding Protein/P300
mTOR	mammalian target of rapamycin
ARD1	arrest defective-1
MTA1	aetastasis-associated protein 1
CDK1	Cyclin-dependent kinase 1
ATM	ataxia and telangiectasia mutated
PLK3	polo-like kinase-3
MTA1	metastasis-associated protein 1
GSK3	glycogen synthase kinase-3
AMPK	adenosine 5'-monophosphate (AMP)-activated protein kinase
MAPK	mitogen-activated protein kinase
HepG2	human liver hepatocellular cell lines
GOLD	global initiative for chronic obstructive lung disease
mMRC	modified Medical Research Council
CC-LR	lung epithelium of the K-ras mutant mice mode
NDRG1	N-myc downstream regulated gene 1
SCLC	small cell lung cancer
SOCS3	suppressors of cytokine signaling 3
ncRNAs	non-coding RNAs
miRNA	microRNA
HPMVECs	human pulmonary microvascular endothelial cells
SIRT1	sirtuin 1
MRC-5	lung fibroblasts
NaHS	sodium hydrosulphide
PDTC	pyrrolidine dithiocarbamate
LPS	lipopolysaccharide
LBP	lycium barbarum polysaccharides
